# Long term complications and vision loss in HLA-B27 uveitis

**DOI:** 10.1038/s41433-022-02216-x

**Published:** 2022-08-29

**Authors:** Haya H. Al-Ani, Joanne L. Sims, Rachael L. Niederer

**Affiliations:** 1 Department of Ophthalmology, Auckland District Health Board, Auckland, New Zealand; 2 Department of Ophthalmology, University of Auckland, Auckland, New Zealand

**Keywords:** Uveal diseases, Risk factors

## Abstract

**Objectives:**

To evaluate the long term complications and vision loss in HLA-B27 uveitis.

**Methods:**

Retrospective review of subjects with HLA-B27 uveitis in a public tertiary centre between January 2008 and 2020.

**Results:**

562 HLA-B27-positive subjects (834 eyes) had mean follow-up of 9.8 years (8173.2 eye-years). Median visual acuity at ten years was 0.1 logMAR (IQR 0.0–0.1). Complications occurred in 404 eyes (48.4%): posterior synechiae (39.7%), cataract (22.1%), elevated intraocular pressure (15.5%), cystoid macular oedema (6.0%). Permanent moderate vision loss ( ≤ 0.4 logMAR) due to uveitis occurred in 14 eyes (1.7%) and severe vision loss (≤ 1.0 logMAR) in 7 eyes (0.8%). Complications were more common with older age (OR 1.017 *p* = 0.016), chronic inflammation (OR 5.272 *p* < 0.001) and intermediate uveitis (OR 5.982 *p* < 0.001).

**Conclusions:**

Complications are frequent in HLA-B27 uveitis, especially in older subjects, chronic inflammation and intermediate uveitis. Despite this, the majority of subjects maintain good visual prognosis.

## Introduction

Initially described in 1973, the association between HLA-B27 and its associated spectrum of inflammatory diseases remains one of the strongest HLA-disease associations known to date [1]. This spectrum of disease includes uveitis, ankylosing spondylitis, reactive arthritis, inflammatory bowel disease and psoriatic arthritis [2–5]. HLA-B27 uveitis regularly presents as isolated uveitis or occasionally scleritis; however, it is frequently the first indication of a previously undiagnosed HLA-B27-associated inflammatory condition, usually a spondyloarthropathy [6].

Anterior uveitis is the most common form of uveitis in HLA-B27-associated disease, accounting for approximately 90% of presentations [7–9]. Conversely, HLA-B27 is the most common identifiable cause of anterior uveitis, representing 18–32% of cases in Western countries [10–16]. Although vision loss in anterior uveitis is uncommon, the prognosis associated with HLA-B27 is less favourable than that in HLA-B27-negative patients [17]. It is especially significant in the working-age population, which is vulnerable to long-term morbidity and reduced productivity [13].

The purpose of this study was to evaluate the long term complications and vision loss in HLA-B27.

## Methods

### Subject selection

Subjects with HLA-B27 uveitis were identified from a database of subjects with uveitis seen in Uveitis Clinic (acute clinic and specialist clinic) at Auckland District Health Board between January 2008 and January 2020. Ethics Committee approval was obtained prior to data collection (Auckland ethics approval AH1339).

### Data collection

Anatomical location of uveitis was defined according to Standardized Uveitis Nomenclature (SUN) criteria [18]. Chronic anterior uveitis (CAU) was defined as persistent anterior uveitis characterized by prompt relapse (in less than 3 months) after discontinuation of therapy [18]. The best corrected visual acuity (BCVA) results were converted to logMAR units for analysis with the following conversion used for vision of counting fingers or worse; counting fingers 2.0 logMAR; hand movements 2.3 logMAR; light perception 2.6 logMAR; no light perception 2.9 logMAR [19]. The outcome of permanent moderate vision loss (MVL; range 0.4–1.0 logMAR) and severe vision loss (SVL ≤ 1.0 logMAR) was defined according to the SUN Working Group [18].

Clinically significant cataract was defined as the presence of 1+ nuclear sclerosis, 1+ cortical cataract, or 0.5+ posterior subcapsular cataract. Cystoid macular oedema was diagnosed by ocular coherence tomography (OCT) or fluorescein angiography. Epiretinal membrane was diagnosed on clinical examination and/or on OCT. Uveitic glaucoma was considered to be glaucomatous optic neuropathy due to raised intraocular pressure in the setting of uveitis in the absence of other causes of glaucoma (such as pre-existing glaucoma, pigment dispersion, normal tension glaucoma, etc). Hypotony was defined as sustained intraocular pressure  ≤6 mmHg.

### Statistical analysis

Data was entered into an Excel spreadsheet and analysed in STATA version 15 (StataCorp 2017, College Station, TX). Categorical variables are presented as n(%) and continuous variables are presented as median (interquartile range [IQR]). Complications were reported as rates per eye [18]. A random-effects logit model with eyes clustered within subjects was used to examine risk of complications. A p value of <0.05 was considered significant.

## Results

During the study period, 2753 subjects with uveitis were reviewed in acute or tertiary uveitis clinics, of whom 562 (20.4%) had HLA-B27-positive uveitis. Disease was unilateral in 288 (51.3%), simultaneous bilateral in 26 (4.6%) and alternating in 248 (44.1%). 834 eyes were included in the analysis. Subject demographics are reported in Table 1. Median age at first presentation with uveitis was 38.0 years, with 91.5% aged 65 years or below, and 347 subjects (61.7%) were male. No difference was observed in age at presentation in males compared to females (*p* = 0.131).

**Table 1:** Subject demographics and clinical presentation.

	*N* = 562 subjects 834 eyes
Age	Median 38.0 (IQR 29.9–50.0)
Male	347 (61.7%)
Ethnicity	
Caucasian	330 (58.7%)
Indian	49 (8.7%)
Other Asian	86 (15.3%)
Maori	42 (7.5%)
Pacific Islander	50 (8.9%)
Other	2 (0.4%)
Unknown	3 (0.5%)
Systemic associations	
Ankylosing spondylitis	180 (32.0%)
Inflammatory bowel disease	17 (3.0%)
Psoriatic arthritis	12 (2.1%)
Reactive arthritis	14 (2.5%)
Laterality	
Unilateral	288 (51.3%)
Simultaneous bilateral	26 (4.6%)
Alternating	248 (44.1%)
Anatomical location	
Anterior	805 (96.5%)
Intermediate	73 (8.8%)
Panuveitis	19 (2.3%)
Scleritis	5 (0.6%)
Chronic inflammation	57 (6.8%)

Median visual acuity at presentation was 0.1 logMAR (IQR 0.0–0.2). Median presenting intraocular pressure (IOP) was 12 mmHg (IQR 10–16). Presenting IOP was ≥ 24 mmHg in 27 eyes (3.2%), including 11 eyes with IOP ≥ 30 mmHg (1.3%). Subjects were more likely to present with IOP ≥ 24 mmHg if they were older (OR 1.073 *p* = 0.005), or had intermediate uveitis (OR 8.449 *p* = 0.010). Patients who were subsequently classified as having chronic uveitis were also more likely to have high presenting IOP (OR 10.925 *p* = 0.023). There was no association with raised presenting IOP and gender, ethnicity or ankylosing spondylitis.

Disease-modifying anti-rheumatic therapy (DMARDs) were used in 31 subjects (5.5%). Anti-tumour necrosis factor (anti-TNF) drugs were used in 38 subjects (6.9%), 13 of whom had combined DMARD and anti-TNF therapy (2.3%). Uveitis control was the indication for DMARD therapy in two subjects; combined DMARD and anti-TNF therapy was used in two subjects for uveitis control. The indication for DMARD or anti-TNF therapy in all other subjects was either spondyloarthropathy or inflammatory bowel disease control.

## Complications

Mean follow-up was 9.8 years (median 7.3 IQR 1.8–12.7, 8173.2 eye-years). Complications occurred in 404 eyes (48.4%). Subject complications are reported in Table 2. Posterior synechiae were the most frequent complication, occurring in 339 eyes (39.7%) followed by cataracts in 183 eyes (22.1%). Five eyes were pseudophakic at presentation. Risk factors for complications are reported in Table 3. On multivariate analysis, older age (OR 1.017 *p* = 0.016), intermediate uveitis (OR 5.273 *p* < 0.001) and chronic inflammation (OR 5.982 *p* < 0.001) were associated with more complications.

**Table 2:** Complications of HLA-B27 uveitis.

	*N* = 834 eyes
Posterior synechiae	330 (39.7%)
Cataract	183 (22.1%)
Cataract surgery	94 (11.3%)
Papillitis	7 (0.8%)
Epiretinal membrane	26 (3.1%)
Cystoid macular oedema	50 (6.0%)
Intraocular pressure ≥24 mmHg	129 (15.5%)
Uveitic glaucoma	28 (3.5%)
Glaucoma surgery	8 (1.0%)
Peripheral iridotomy	14 (1.7%)
Hypotony	4 (0.5%)
Retinal detachment	3 (0.4%)
Retinal vasculitis	6 (0.7%)
Moderate visual loss (6/15–6/60)	13 (1.6%)
Severe visual loss (≤ 6/60)	7 (0.8%)

**Table 3:** Risk factors for developing complications.

	Univariate		Multivariate	
	OR	*P*-value	OR	*P*-value
Age	1.020	0.006	1.017	0.016
Female	0.952	0.821		
Maori/Pasifika	1.002	0.278		
Intermediate uveitis	6.466	<0.001	5.272	<0.001
Chronic inflammation	8.018	<0.001	5.982	<0.001
Ankylosing spondylitis	0.970	0.891		
Inflammatory bowel disease	0.305	0.078	0.366	0.105
Psoriatic arthritis	0.999	0.577		
Reactive arthritis	0.990	0.769		

Median visual acuity at one year was 0.0 logMAR (IQR 0.0–0.1 *n* = 509 eyes), at five years was 0.0 logMAR (IQR 0.0–0.1 *n* = 346 eyes) and at ten years was 0.1 logMAR (IQR 0.0–0.1 *n* = 187 eyes). MVL occurred in 35 eyes (4.2%), of which 13 (1.6%, rate 0.0016/eye-year) had permanent MVL secondary to uveitis [Table 4]. The most common causes of permanent MVL secondary to uveitis were uveitic glaucoma in 5 eyes and epiretinal membrane in 3 eyes. Other causes (either non-uveitic or non-permanent) included active inflammation or corneal oedema at last review in 12 eyes, where vision loss was not felt to be permanent, and cataract in 10 eyes. Three eyes had pre-existing causes of vision loss: amblyopia 1 eye, keratoconus 1 eye, and retinal dystrophy 1 eye. SVL occurred in 8 eyes (1.0%) of which 7 (0.8%, rate 0.0008/eye-year) had permanent SVL due to uveitis with the most common cause being uveitic glaucoma in 3 eyes [Table 5]. Other causes included a macular hole in 1 eye.

**Table 4:** Causes of permanent moderate vision loss (MVL) secondary to uveitis.

Cause of MVL	*N* = 13 eyes
Uveitic glaucoma	5 (38.5%)
Epiretinal membrane	3 (23.0%)
Retinal detachment	1 (7.7%)
Suprachoroidal haemorrhage post-op	1 (7.7%)
Cystoid macular oedema	1 (7.7%)
Hypotony	1 (7.7%)
Corneal scar	1 (7.7%)

**Table 5:** Causes of severe vision loss (SVL) secondary to uveitis.

Cause of SVL	*N* = 7 eyes
Uveitic glaucoma	3 (42.8%)
Pupillary membrane	1 (14.3%)
Cystoid macular oedema	1 (14.3%)
Choroidal neovascular membrane	1 (14.3%)
Enucleation for severe uveitic glaucoma	1 (14.3%)

Posterior synechiae occurred in 330 eyes (39.7%). Posterior synechiae were more likely to occur in older subjects (OR 1.017 *p* = 0.036), chronic inflammation (OR 3.518 *p* = 0.004) and in eyes with intermediate uveitis (OR 4.902 *p* < 0.001). There was no association with gender, ethnicity or with ankylosing spondylitis. Subjects with posterior synechiae were more likely to develop cystoid macular oedema (OR 4.904 *p* = 0.001), epiretinal membrane (OR 7.270 *p* = 0.003), or raised intraocular pressure (OR 3.929 *p* < 0.001). There was no increased rate of cataract associated with posterior synechiae. YAG or surgical peripheral iridotomy was required in 10 eyes (1.2%) for iris bombé.

Cataract occurred in 183 eyes (22.1%). Cataract was more likely with older age at presentation (OR 1.346 *p* < 0.001), chronic inflammation (OR 11.189 *p* < 0.001) and intermediate uveitis (OR 2.189 *p* < 0.001). There was no association between cataract and gender, ethnicity or ankylosing spondylitis. Cataract surgery was performed in 94 subjects with a median age of 63.9 years (IQR 47.8–72.4). Cataract surgery was performed under the age of 65 years for 68 subjects (72.3%). Median duration of uveitis prior to cataract surgery was 5.9 years (IQR 2.2–14.0).

Raised intraocular pressure occurred in 129 subjects (15.5%). It was more common in chronic uveitis (OR 31.7 *p* < 0.001) and intermediate uveitis (OR 6.264 *p* = 0.001). There was no association with age, gender, ethnicity or ankylosing spondylitis.

Cystoid macular oedema (CMO) occurred in 50 eyes (6.0%). There was no association with age, gender or ethnicity and odds of developing CMO. Subjects were more likely to develop CMO if they had chronic inflammation (OR 15.744 *p* = 0.004) or intermediate uveitis (9.870 *p* = 0.005). Subjects with ankylosing spondylitis had more CMO but this did not reach statistical significance on univariate analysis (OR 2.670 *p* = 0.072). Exploring this further with multivariate analysis controlled for chronic inflammation and intermediate uveitis, there was a significantly higher rate of CMO in subjects with ankylosing spondylitis (OR 2.896 *p* = 0.048). The median time between uveitis flare and CMO development was 24 days (IQR 14–40). Topical therapy alone was used in 33 eyes (66%) with subsequent escalation of steroid therapy required in 28 eyes (56%): oral prednisone in 9 eyes, subTenons steroid in 7 eyes, orbital floor steroid in 11 eyes, and intravitreal triamcinolone in 1 eye. The initial episode of CMO resolved in 49 eyes of which 14 had at least one recurrence. Intermediate uveitis developed in 13 eyes with CMO (26.0%), the majority (11/13; 85%) occurring at the time of CMO diagnosis.

Epiretinal membrane occurred in 26 subjects (3.1%) and was associated with chronic inflammation (OR 7.240 *p* = 0.003) and intermediate uveitis (OR 7.515 *p* = 0.001). There was no association with age, gender, ethnicity or ankylosing spondylitis.

## Discussion

HLA-B27-associated uveitis accounted for one-fifth of uveitis presentations to acute or specialist clinics in our cohort. The majority of the affected population (91.5%) was working age (≤ 65 years) at presentation, with a male preponderance, and anterior uveitis accounting for more than 96% of these presentations. Complications occurred in 48.4% and were more common in subjects who were older at presentation, had chronic inflammation or intermediate uveitis. Despite this, vision was maintained throughout the long follow-up of this study, with only 2.5% developing permanent vision loss due to uveitis.

HLA-B27 uveitis is generally known as a condition of the young, with a median age of onset in the 30 s [9, 16, 20, 21]. Young age is also a significant risk factor in ankylosing spondylitis, the most common associated spondyloarthropathy, presenting most frequently in 20–30 years old [22]. In the current study, older age was significantly associated with developing complications. There have been cases described of older individuals presenting in their seventh or eighth decade of life with severe inflammation and hypopyon. The underlying pathophysiology is unclear; however, despite this severity, the inflammation could be managed adequately with prolonged topical anti-inflammatory therapy [23].

Chronic inflammation is known to increase complication rates in anterior uveitis [13, 24], and this was reflected in our cohort as well. Chronic anterior uveitis is a significant risk factor for development of uveitic glaucoma, CMO, and epiretinal membrane, accounting for a higher risk of vision loss compared to acute inflammation [13]. When occurring in HLA-B27 anterior uveitis, chronic inflammation is seen more commonly with psoriatic arthritis, while it is not usually associated with inflammatory bowel disease or reactive arthritis [22]. The extra-ocular diagnosis is usually made following a diagnosis of uveitis, so systemic screening questions and subsequent relevant investigations are an important aspect of the assessment at presentation with uveitis.

Intermediate uveitis is a less common manifestation of HLA-B27 ocular disease, and results in higher rates of complications compared to anterior uveitis. In general, the disease course with intermediate uveitis is prolonged with frequent complications, such as CMO. While the long-term visual prognosis is generally good, patients often require systemic steroid and/or other immunosuppressant treatment to manage the inflammation [25]. We recommend intensive initial therapy and close monitoring for complications in these cases.

Formation of posterior synechiae is a result of intraocular inflammation in the aqueous and is the most common complication in HLA-B27 uveitis [6, 17, 26]. However, the rates of posterior synechiae vary widely in the literature with a range of 13.1–55%, which is likely due to the different cohort sizes and duration of follow-up [6, 8, 9, 17, 26]. Our cohort’s rate of almost 40% (0.0404/eye-year) is in the higher end of this range. Previous reports recognise chronic inflammation and recurrence of uveitis to be associated with posterior synechiae [6, 9, 27]. Our findings contrast with Tay-Kearney et al. [9], as our study did not observe increased cataract formation in the presence of posterior synechiae. Of significance, the development of posterior synechiae can result in seclusion papillae and subsequent iris bombé. In our study, peripheral iridotomy was required in 1.2% of eyes to relieve iris bombé. Subjects with uveitis have a high risk of failure with YAG peripheral iridotomy and surgical iridotomy is recommended [28]. Intensive anti-inflammatory therapy in active uveitis, along with topical mydriatics, is essential for preventing and breaking the formation of posterior synechiae [29].

Cataract development was common within our study, developing in 22.1% with increased risk in older subjects, chronic inflammation and intermediate uveitis. Median age at time of cataract surgery was 63.9 years, with the majority (72.3%) being done at age ≤ 65years. In pre-presbyopic individuals, careful discussion around refractive targets and reading vision are required. Development of cataract in uveitis may be a complication of inflammation or occur secondary to steroid use. There is conflicting evidence on whether cataract formation risk is higher in HLA-B27-positive uveitis, but there was no significant difference in meta-analysis results [29]. We did not identify any published study specific to outcomes of cataract surgery in HLA-B27 uveitis. However, a recent large study demonstrated uveitic eyes had smaller pupil size, increased additional procedures, more intraoperative complications, and poorer postoperative visual acuity up over a six month follow-up period, compared to non-uveitic eyes undergoing cataract surgery [30]. Pre-operative risk factor assessment is essential for discussing prognosis with patients and minimising complication risks where possible.

CMO developed in 6% of our cohort, which is in the lower range of the reported 2.4–30% of HLA-B27-positive cases [17, 21]. In a comparison between HLA-B27-positive and -negative patients with anterior uveitis, CMO was found to be five times more frequent in HLA-B27-associated disease, corresponding to an eightfold increase in the requirement for systemic steroids in this group [17], although this was not reflected in a subsequent meta-analysis [30]. Chronic inflammation and intermediate uveitis were risk factors for CMO, and a quarter of the CMO cases in our cohort developed intermediate uveitis. Considerable variability occurred in treatment algorithms for HLA-B27 CMO within our cohort. With the recently published findings of the POINT study, it appears that intravitreal triamcinolone or intravitreal dexamethasone implant are superior to periocular triamcinolone in treating uveitic macular oedema, with a modest increase in risk of intraocular pressure rise [31]. Anterior uveitis constituted a small proportion of this study, so further randomised controlled trials are important to guide management in this group, particularly those with chronic inflammation.

Epiretinal membrane (ERM) features less frequently in the literature on complications in HLA-B27 uveitis and it is certainly a less common complication in our cohort. Risk factors for ERM development include chronic inflammation, Asian ethnicity, increasing age, and diabetes [13, 32, 33]. Despite occurring infrequently, it is important to acknowledge the role of ERM in vision loss. It is particularly significant in the context of CMO, where it has been shown to decrease the likelihood of CMO resolution and subsequently reduce visual acuity [34].

Glaucomatous optic neuropathy was uncommon in our series, occurring in 3.8% of eyes. A recent study of over 2500 eyes demonstrates that glaucoma is the most frequent cause of permanent vision loss in anterior uveitis [13]. Raised intraocular pressure was very frequent within our series, and is amongst the most common reported complications of HLA-B27 uveitis [24]. Raised intraocular pressure in HLA-B27 uveitis may arise secondary to acute inflammation and/or inflammatory debris, secclusio pupillae leading to iris bombé, development of peripheral anterior synechiae, or steroid response. We recommend baseline documentation of the optic nerve and yearly nerve assessment and gonioscopy as well as assessment of intraocular pressure at each review.

Limitations of this study include the retrospective nature and reliance on documentation of clinical findings. The strengths include inclusion of a large number of subjects from both acute and tertiary clinics, collection of data by direct review of the clinical notes rather than electronic databases, and the long duration of follow-up.

This study demonstrates that the complication rate was high in HLA-B27 uveitis, however, the visual prognosis remained good with median BCVA of 0.1 at ten years. Subjects were more likely to develop complications if they were older, had chronic inflammation or intermediate uveitis. Further research evaluating the risk factors for recurrences are needed, along with further randomised controlled trials to optimise management of CMO in anterior uveitis.

### Summary

#### What was known before

- HLA-B27 is a common cause of anterior uveitis.
- Visual prognosis in HLA-B27 uveitis is less favourable compared to HLA-B27 negative cases.

#### What this study adds

- HLA-B27 complications are associated with with older age, chronic inflammation, and intermediate uveitis.
- The most common complications were posterior synechiae, cataract, raised intraocular pressure, and cystoid macular oedema.
- The majority of HLA-B27 uveitis cases have a good visual outcome.

## Data Availability

The datasets generated during and/or analysed during the current study are available from the corresponding author on reasonable request.
